# Structural and functional brain alterations in patients with myasthenia gravis

**DOI:** 10.1093/braincomms/fcac018

**Published:** 2022-02-01

**Authors:** Benita Klaus, Patrick Müller, Nora van Wickeren, Milos Dordevic, Marlen Schmicker, Yael Zdunczyk, Tanja Brigadski, Volkmar Leßmann, Stefan Vielhaber, Stefanie Schreiber, Notger G. Müller

**Affiliations:** German Centre for Neurodegenerative Diseases, 39120 Magdeburg, Germany; Department of Neurology, Otto-von-Guericke University, 39120 Magdeburg, Germany; German Centre for Neurodegenerative Diseases, 39120 Magdeburg, Germany; Department of Neurology, Otto-von-Guericke University, 39120 Magdeburg, Germany; German Centre for Neurodegenerative Diseases, 39120 Magdeburg, Germany; Department of Neurology, Otto-von-Guericke University, 39120 Magdeburg, Germany; German Centre for Neurodegenerative Diseases, 39120 Magdeburg, Germany; Department of Neurology, Otto-von-Guericke University, 39120 Magdeburg, Germany; German Centre for Neurodegenerative Diseases, 39120 Magdeburg, Germany; Department of Neurology, Otto-von-Guericke University, 39120 Magdeburg, Germany; Institute of Physiology, Otto-von-Guericke University, 39120 Magdeburg, Germany; Department of Informatics and Microsystems Technology, University of Kaiserslautern, 67659 Zweibrücken, Germany; Institute of Physiology, Otto-von-Guericke University, 39120 Magdeburg, Germany; Center for Behavioral Brain Sciences (CBBS), 39120 Magdeburg, Germany; German Centre for Neurodegenerative Diseases, 39120 Magdeburg, Germany; Department of Neurology, Otto-von-Guericke University, 39120 Magdeburg, Germany; Center for Behavioral Brain Sciences (CBBS), 39120 Magdeburg, Germany; German Centre for Neurodegenerative Diseases, 39120 Magdeburg, Germany; Department of Neurology, Otto-von-Guericke University, 39120 Magdeburg, Germany; Center for Behavioral Brain Sciences (CBBS), 39120 Magdeburg, Germany; German Centre for Neurodegenerative Diseases, 39120 Magdeburg, Germany; Department of Neurology, Otto-von-Guericke University, 39120 Magdeburg, Germany; Faculty of Health Sciences, University of Potsdam, 14476 Potsdam, Germany

**Keywords:** myasthenia gravis, neuroplasticity, VBM, neuropsychological testing, BDNF

## Abstract

Myasthenia gravis is an autoimmune disease affecting neuromuscular transmission and causing skeletal muscle weakness. Additionally, systemic inflammation, cognitive deficits and autonomic dysfunction have been described. However, little is known about myasthenia gravis-related reorganization of the brain. In this study, we thus investigated the structural and functional brain changes in myasthenia gravis patients. Eleven myasthenia gravis patients (age: 70.64 ± 9.27; 11 males) were compared to age-, sex- and education-matched healthy controls (age: 70.18 ± 8.98; 11 males). Most of the patients (*n* = 10, 0.91%) received cholinesterase inhibitors. Structural brain changes were determined by applying voxel-based morphometry using high-resolution T_1_-weighted sequences. Functional brain changes were assessed with a neuropsychological test battery (including attention, memory and executive functions), a spatial orientation task and brain-derived neurotrophic factor blood levels. Myasthenia gravis patients showed significant grey matter volume reductions in the cingulate gyrus, in the inferior parietal lobe and in the fusiform gyrus. Furthermore, myasthenia gravis patients showed significantly lower performance in executive functions, working memory (Spatial Span, *P* = 0.034, *d *= 1.466), verbal episodic memory (*P* = 0.003, *d* = 1.468) and somatosensory-related spatial orientation (Triangle Completion Test, *P* = 0.003, *d *= 1.200). Additionally, serum brain-derived neurotrophic factor levels were significantly higher in myasthenia gravis patients (*P *= 0.001, *d *= 2.040). Our results indicate that myasthenia gravis is associated with structural and functional brain alterations. Especially the grey matter volume changes in the cingulate gyrus and the inferior parietal lobe could be associated with cognitive deficits in memory and executive functions. Furthermore, deficits in somatosensory-related spatial orientation could be associated with the lower volumes in the inferior parietal lobe. Future research is needed to replicate these findings independently in a larger sample and to investigate the underlying mechanisms in more detail.

## Teaser

Klaus *et al*. compared myasthenia gravis patients to matched healthy control subjects and identified functional alterations in memory functions as well as structural alterations in the cingulate gyrus, in the inferior parietal lobe and in the fusiform gyrus.

## Graphical Abstract

**Graphical Abstract:**
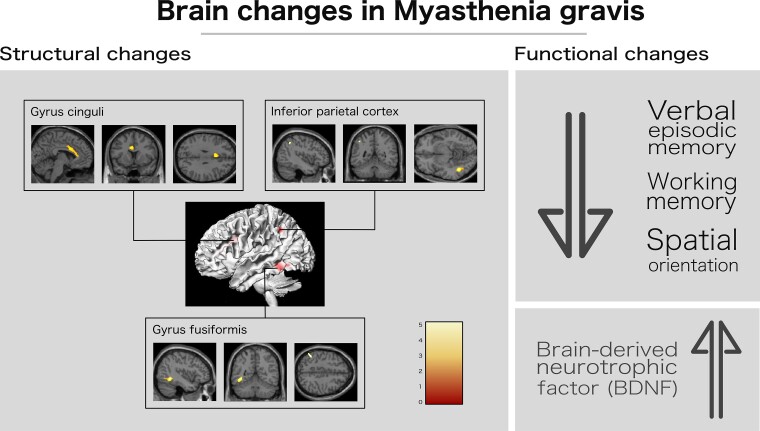

## Introduction

Myasthenia gravis (MG), a chronic peripheral neuromuscular autoimmune disease, is characterized by skeletal muscle weakness, systemic inflammation and autonomic dysfunction.^1^ Characteristically, the peripheral symptoms such as skeletal muscle weakness increase during the day and first manifest in the external ocular muscles; smooth and cardiac muscles are not affected.^1^ In addition, several studies have described cognitive deficits, especially affecting verbal and visual learning, processing speed, reaction time and memory.^2^ Additionally, Sabre *et al.*^3^ demonstrated significantly poorer strategic solution planning and reduced exploration time in autoantibody positive mice. In contrast, Feldmann *et al.*^4^ reported no significant differences in memory, attention and intelligence. They hypothesized that the reported poorer measurements were due to sleepiness during the day and exhaustibility. However, the structural brain changes presumed to underlie cognitive dysfunction are not sufficiently understood.^5,6^ The current study thus aims to not only investigate cognitive function and plasticity in MG, but also intends to assess the related structural grey matter brain changes.

There are at least two potential ways in which MG might affect the CNS, and thus cognition. Firstly, MG autoantibodies may have central cholinergic effects that result in cognitive dysfunction, especially memory functions.^3,6,7^

The second mechanism that may lead to CNS effects in MG is somatosensory deprivation. Recent studies indicate that somatosensory-enriched environment and intensive training can enhance brain plasticity.^8,9^ Vice versa, an extreme form of sensorimotor deprivation, arm amputation, has been shown to result in a reduction in volume of the somatosensory cortex.^10^ Volume reductions were also observed after nerve damages to certain fingers. Corresponding to the reductions in the corresponding areas of the damaged fingers, the areas of the healthy fingers increased in volume.^11^ Therefore, we hypothesize that reduced physical activity in MG due to muscle weakness could result in less sensorimotor and affect brain plasticity negatively.

In this study, we investigate structural and functional brain changes in MG patients compared to age, gender and education-matched healthy controls applying a broad battery of cognitive, somatosensory tests, MRI and brain-derived neurotrophic factor (BDNF) blood samples. We hypothesized that MG patients will not only perform worse in several cognitive tests, but also in functions based on somatosensory input. We expected that these deficits will be accompanied by specific alterations in structural brain plasticity. Understanding potential brain alterations in MG can enhance our knowledge of the pathophysiological mechanisms of MG and help to develop tailored interventions to counteract cognitive deficits.

## Materials and methods

### Study design and participants

The study was designed as a cross-sectional pilot study and approved by the ethics committee at the Otto-von-Guericke University Magdeburg (Germany). All participants signed a written informed consent form prior to participation. MG patients were recruited from the clinic for neurology. Healthy controls were recruited by advertisements in local newspapers and public notices.

**Figure 1:**
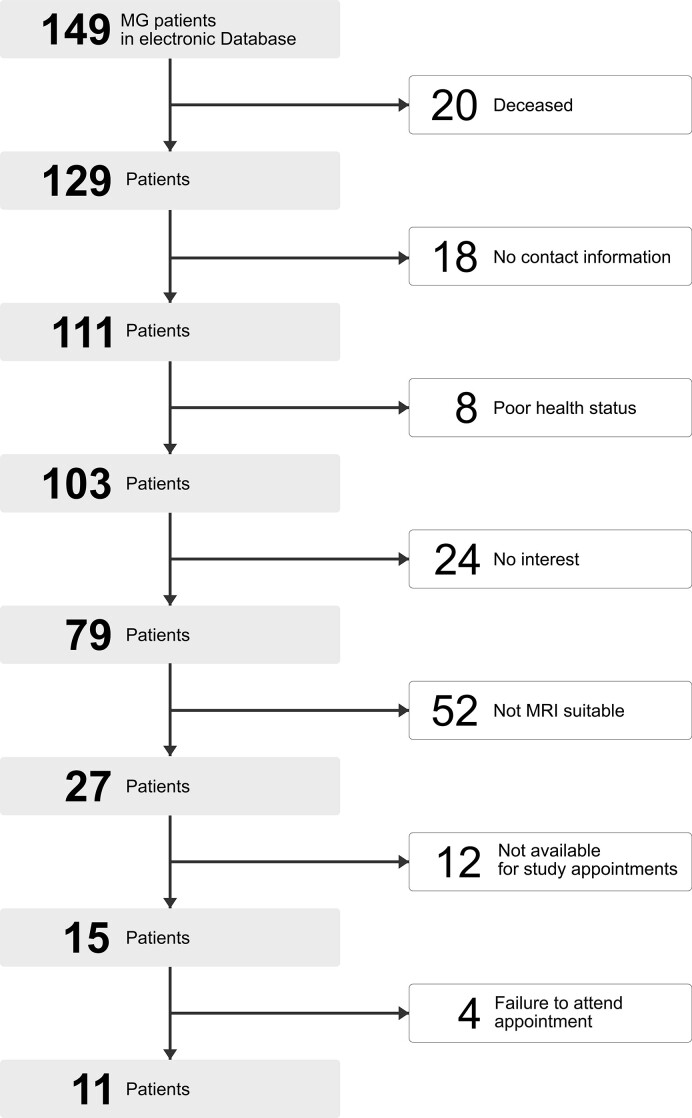
Reasons for exclusion.

Patients with generalized, mild-to-moderate late-onset MG (LOMG; Myasthenia Gravis Foundation of America II–III) diagnosed between 2001 and 2017 were selected from an electronic patient database. LOMG is currently defined in the literature with an age of onset between 45 and 70 years.^12–14^ Further inclusion criteria involved right handedness, normal or corrected-to-normal vision and no neurovascular, neurodegenerative or further neuroimmunological disorders or diagnoses [i.e. dementia (Mini Mental State Examination, MMSE < 27) and depression (Beck’s Depression Inventory, BDI-II > 18)]. Based on these criteria, 149 patients were identified as eligible for the study. Of these, 38 could not be reached or were already deceased, 24 did not wish to participate and 52 had contraindications against 3 T MRI. Reasons for exclusion also included no availability in terms of capacities, failure to attend appointments or poor health status (Fig. 1).

After screening, 11 MG patients (age: 70.64 ± 9.27; 11 males) could be recruited for this study and compared to age-, gender- and education-matched healthy controls (age: 70.18 ± 8.98; 11 males; Table 1). Age (*P *= 0.869) and years of education (*P *= 0.527) did not differ significantly between patients and healthy controls. Under the current medication, MG patients did not show relevant subjective and objective neurological deficits, e.g. in testing for degrees of strength and pallesthesia. The tests were performed according to a standardized procedure and within 1 day to avoid sequence effects. All screening tools and questionnaires were given out in the validated German translation.

**Table 1:** Patients and healthy controls

Controls			Patients							
Gender	Age	Ed^a^	Gender	Age	Ed	MGFA^b^	Duration	Medication	AAb^c^	Besinger
M	56	14	M	54	13	IIb	70.50	Mycophenolat	AChR	0.88
M	53	12	M	55	12	IIa	44.50		AChR	n.a.
M	66	16	M	65	16	IIa	38.00	Pyridostigmin, Azathioprin	AChR	0.5
M	69	13	M	69	15	IIb	41.50	Pyridostigmin, Prednisolon	AChR	1.2
M	66	15	M	69	15	IIb	52.00	Pyridostigmin, Azathioprin	AChR	n.a.
M	73	17	M	75	18	IIb	12.00	Mycophenolat, Pyridostigmin, Prednisolon	AChR	n.a.
M	74	17	M	76	16	IIa	202.50	Azathioprin	AChR	n.a.
M	79	13	M	77	12	IIIb	23.50	Azathioprin, Prednisolon	AChR	1.45
M	78	13	M	78	11	IIb	35	Azathioprin, Pyridostigmin	AChR	1.2
M	79	11	M	79	11	IIa	10.50	Azathioprin, Prednisolon, Pyridostigmin	AChR	1.2
M	78	15	M	80	13	IIa	80.00	Pyridostigmin	AChR	n.a.

Gender, age, years of education, disease duration till the study date (from onset of symptoms; in month), Myasthenia Gravis Foundation of America, medication, autoantibody status, Besinger-Score. Source: Osserman and Genkins.^70^

^a^ Years of education.

^b^ Myasthenia Gravis Foundation of America.

^c^ AAb = autoantibody status.

### Clinical measurements

The clinical measurements included muscle strength tests according to the Medical Research Council (MRC) and pallesthesia (vibration sensation).^15,16^

### Screening tools

#### Dementia screening

For early dementia detection, the MMSE was used, which is standardized in dementia screening and follow-up.^17^ Scores below 27 would have been indicative of dementia and resulted in the exclusion from this study.^18^

#### Depression screening

The BDI, a ‘self-report instrument to assess the severity of depressive symptomatology’, was applied for the exclusion of a relevant depression.^19^ A score of 18 or more is interpreted as clinically relevant depression and resulted in the exclusion from this study.

### Questionnaires

#### Leisure interests

For a more detailed assessment of the subjects’ leisure time, the questionnaire for leisure interests was completed. This questionnaire records the leisure interests of adults in detail and thus provides an insight into the mental and social demands to which the participant is exposed.^20^

#### Fatigue

The Fatigue Severity Scale is designed to help assess the subjectively perceived and vague problem of fatigue and to evaluate its severity.^21^

### Cognitive testing—general

An extensive battery of neuropsychological testing consisted of six measurements.

#### Verbal learning and short- and long-term memory

The ‘Verbal learning and short- and long-term memory test’ is the German adaptation of the ‘Rey Auditory Verbal Learning Test’ (AVLT), a test that assesses verbal short- and long-term memory.^22,23^ It assesses the different performances of episodic verbal memory; on the one hand, the immediate memory span and on the other hand the recall after a time delay.^24^ There were two lists of 15 words, and each list was repeatedly read to the participant. After each reading, the participant was asked to recall the words. The first list was read out five times, followed by a single reading of the second list. Immediately as well as after a delay of 20 min, the participant was asked to recall the first list from memory. The number of correctly repeated words after each repetition (‘total output’), the recall after a delay of time (‘delayed recall’) and the recognition performance (‘recognition’) were evaluated.

#### Working memory

The digit spam memory test is an important test of working memory. It tests auditory short-term memory, concentration ability and reversibility.^25^ Sequences of numbers with ascending number length were read out to the participant, who then had to repeat them. The number sequences successfully repeated were scored.

#### Visual–verbal performance

The Stroop effect measures the information processing abilities of the visual–verbal domain and the concentrative resistance to automated responses (selectivity), the speed of naming (nomination) and information processing (alertness).^26,27^

#### Word fluency

The Regensburg Word Fluency Test tests the different aspects of word fluency through several subtests. The number of words produced, and the repetition errors were evaluated.^27^

#### Visuospatial working memory

Visuospatial Working Memory was assessed via the Spatial Span (SSP), a subtest of the Cambridge Neuropsychological Test Automated Battery (CANTAB). A monitor displayed several white squares. Initially, two of the squares lit up. The participant then had to reproduce the lit-up squares in the correct order by clicking on them. The number of squares increased during the test. The maximum number of correctly reproduced squares and the span length were evaluated.^28^

#### Executive working memory

The One Touch Stockings, a subtest of the CANTAB, assess participants’ executive function, spatial planning and working memory. Based on the principle of the game ‘The towers of Hanoi’, the total time required to complete all tasks was measured.^28,29^

#### Attention ability

The Test Battery for Attention Assessment examines fatigability, attentional performance, response control and responsiveness.^30^ In this study, subjects were tested in three subtests: alertness, divided attention, go/nogo.

### Cognitive testing—spatial orientation

Spatial orientation was assessed for the subfunctions path integration and Rotational Memory (RM) using the Distance Perception (DP), Triangle Completion Test (TCT) and the RM, and the C-Screen, respectively.

#### Path integration/spatial navigation

**Figure 2:**
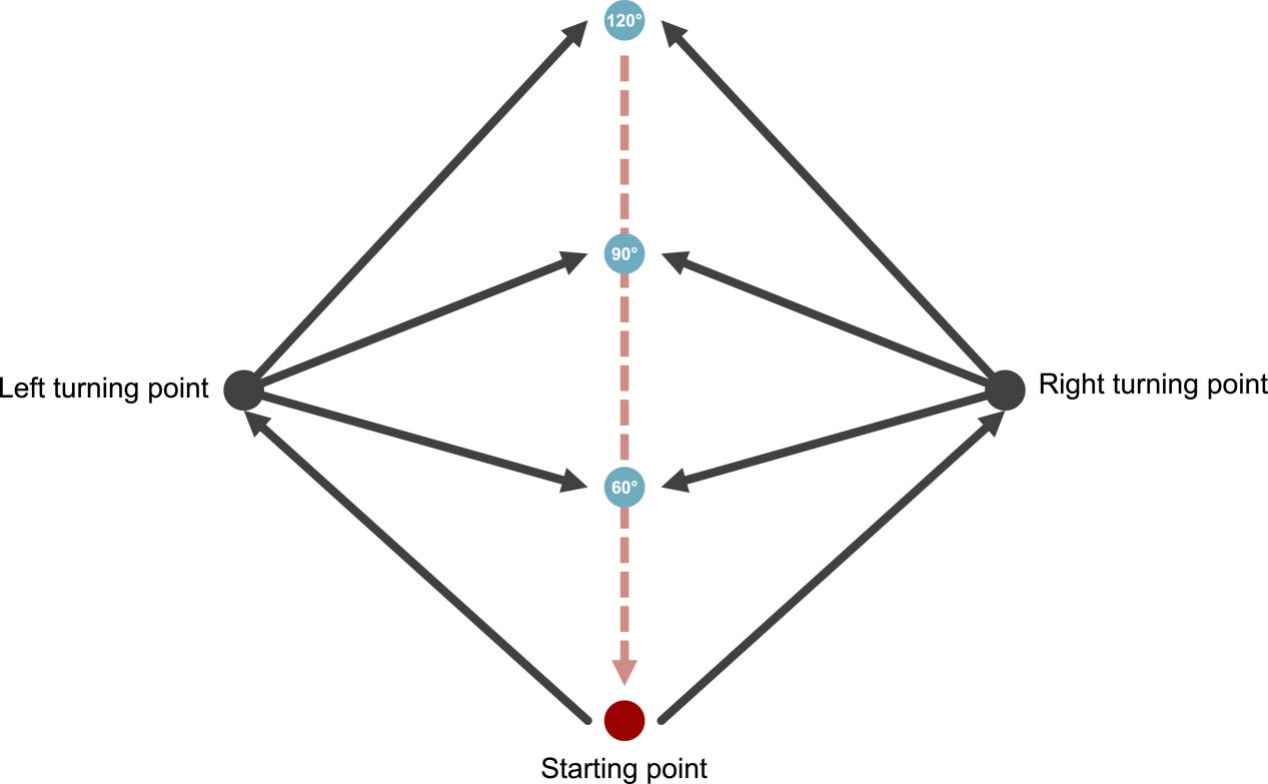
Triangle Completion Test.

To assess non-visual spatial orientation, patients were tested in two measurements. First, with the DP. The participant was blindfolded and led from a fixed starting point 2, 2.5 and 3 m away. From these points, he was asked to find his way back to the starting point on his own. The distance between the presumed starting point and the actual starting point was measured after each test. Secondly, the TCT was performed to assess the vestibular- as well as the somatosensory-related spatial orientation. From a fixed starting point, six triangular paths were marked on the floor. Three on the right and three on the left with angles of 60°, 90° and 120°. The blindfolded participant was first guided along the right and left paths (active; somatosensory-related spatial navigation) and then pushed along the same paths in a wheelchair (passive; vestibular-related spatial navigation). This resulted in a total of 12 trials per participant (three to the right and three to the left, times two conditions). After each path, the participant who was still blindfolded was asked to walk back to the starting point (Fig. 2). The distance between the assumed starting point and the actual starting point was measured after each trial.^31^

#### Rotational memory

For the assessment of the vestibular system, the blindfolded and hearing-protected participant was placed on a test chair. This test chair could rotate in a horizontal direction around its own axis (Interacoustics, Denmark). The participant automatically turned a set number of rotations from a fixed starting point in one or both directions (setup: twice one, two, four and eight rotations). After each rotation pattern, the test leader, following instructions from the participant, turned the participant manually back to its presumed starting point. The angular distance from the patient’s presumed starting point to the actual starting point was evaluated.^32^

#### Spatial orientation

The subjects’ orientation ability was assessed using the C-Screen. The participant was guided through a virtual city and then actively tested for orientation by having to reproduce the route on the one hand and being tested for selective orientation on the other.^33^

### Brain-derived neurotrophic factor

Fasting blood samples were taken in the mornings before the neuropsychological and spatial orientation task assessments. From the blood samples, serum concentrations of BDNF were determined by sandwich ELISAs (BDNF DuoSets; R&D Systems, Wiesbaden, Germany) as previously described.^34^

### MRI acquisition and data analysis

Three Tesla (T) magnetic resonance (MR) images were acquired on a MAGNETOM Skyra^fit^ Scanner (Siemens Healthcare, Erlangen, Germany) using a 32-channel head coil. High-resolution T_1_-weighted magnetization prepared rapid acquisition with gradient echoes sequences were acquired using a 3D magnetization-prepared rapid gradient echo imaging protocol (224 sagittal slices, voxel size: 0.8 mm × 0.8 mm × 0.8 mm, TR: 2500 ms, TE: 3.47 ms, TI: 1100 ms, flip angle: 7°).

The MR images were analysed using voxel-based morphometry (VBM) implemented in SPM 12 (Welcome Department of Cognitive Neurology, London, UK). VBM is a whole-brain unbiased technique for the analysis of regional grey matter volume and tissue changes.^35^ The pre-processing involved grey matter segmentation, template creation via DARTEL, spatial normalization to standardized Montreal Neurological Institute space and smoothing with a Gaussian kernel of 8 mm full width at half maximum.

### Statistical analysis

Statistical analysis of behavioural and clinical data was performed with SPSS 19 (SPSS Inc., Chicago, IL, USA) and R 4.0.3. The data were neither blinded nor randomized. With data normally and homogeneously distributed, the unpaired *t*-test and with data not normally and not homogeneously distributed, the Mann–Whitney U-test was used (with the Bonferroni adjustment). The specified alpha level (*P*) was 0.05. Figures and tables present the effect sizes (Cohen’s *d*) and the respective means with 95% CIs of the difference. Subsequently, Pearson correlation analyses were performed between the significant (regional) grey matter volume differences and significant cognitive testing data. Group differences in grey matter volume were analysed using *t*-test including age and education as covariate. Thereby, a threshold of *P *≤ 0.001 (uncorrected) was applied for all analyses.

### Data availability

Raw data were generated at the German Centre for Neurodegenerative Diseases Magdeburg, Germany. All data supporting the findings of this study are available from the corresponding author on request.

## Results

### Clinical testing

Strength levels (according to the MRC; Table 2) and pallesthesia (peripheral vibration sensation; Table 2) did not show a pathological finding.

**Table 2:** Strength levels

Patient	Age	MRC score	MRC sum score	Scale	Average score	Scale
1	78	209.00	49.76	5	7	3
2	65	210.00	50.00	5	7	3
3	69	208.50	49.64	5	8	3
4	80	208.00	49.52	5	8	3
5	79	202.00	48.10	5	8	3
6	77	206.00	49.05	5	7	3
7	54	210.00	50.00	5	8	3
8	75	207.00	49.29	5	8	3
9	76	210.00	50.00	5	8	3
10	55	189.00	45.00	3	8	3
11	69	210.00	50.00	5	8	3

According to the MRC scale standard. Total score (max. 210 points), MRC formula result (max. 50 points) and associated scale (1—lowest to 5—highest). Pallesthesia scale of patients. Age of patients (in years), average score achieved and associated scale (1—lowest to 3—highest).

### Screening tools

MMSE (*P *= 0.333, *d* = 0.330) and BDI (*P *= 0.649, *d* = 0.207) did not show a significant difference between MG patients and healthy controls. MG patients as well as healthy controls scored between 28 and 30 on the MMSE.

### Questionnaires

The questionnaires for leisure interests (*P* = 0.333, *d* = 0.588 and *P* = 0.695, *d *=* *0.234) as well as for fatigue (*P* = 0.244, *d* = 0.537) did not show a significant difference between MG patients and healthy controls.

### Cognitive testing—general

Statistical analysis revealed significant deficits in visuospatial and verbal working memory [AVLT (variable total output) and CANTAB SSP (variable length)] in MG patients compared to controls. There were no group differences in long-term memory, visual–verbal performance and word fluency revealed (Table 3).

**Table 3:** Results of cognitive testing—general

Cognitive domain	Test	Condition		Patients	Controls	*P*-value	Effect size (d)
Verbal memory	AVLT^a^	Total output^b^		38.6 (8.99)	52.1 (9.40)	0.003	1.468
		Delayed recall^b^		2.45 (1.51)	2.00 (2.19)	0.578	0.239
		Recognition^b^		0.22 (4.63)	1.11 (4.17)	0.958	0.202
Working memory	Digit span	Score^b^		16.6 (5.87)	20.7 (4.08)	0.074	0.811
Visual–verbal performance	Stroop effect 1	Time^c^		19.5 (3.60)	17.5 (2.80)	0.138	0.650
	Stroop effect 2	Time^c^		24.0 (5.60)	23.0 (1.80)	0.789	0.410
	Stroop effect 3	Time^c^		44.5 (17)	43.0 (13.7)	0.569	0.270
	Stroop effect 2	Error^c^		0.00 (75)	0.00 (0.00)	0.549	0.330
	Stroop effect 3	Error^c^		0.00 (2.50)	0.50 (1.00)	0.933	0.330
Word fluency	RWT^d^ FL^e^	Total output^b^		15.6 (6.53)	18.5 (4.93)	0.253	0.501
	RWT FL	Repetition^c^		1.00 (2.00)	1.00 (1.50)	0.832	0.800
	RWT S^f^	Total output^b^		26.1 (6.59)	29.6 (4.59)	0.161	0.616
	RWT S	Repetition^c^		0.50 (1.75)	0.50 (1.00)	0.743	0.210
	RWT FLW^g^	Total output^b^		14.9 (3.99)	15.5 (3.98)	0.712	0.151
	RWT FLW	Repetition^c^		1.00 (1.00)	1.00 (1.00)	0.869	0.000
	RWT SW^h^	Total output^b^		18.5 (3.70)	20.4 (4.18)	0.293	0.481
	RWT SW	Repetition^c^		0.00 (1.00)	1.00 (2.00)	0.150	0.680
Visuospatial working memory	CANTAB^i^ SSP^j^	Score^b^		4.83 (0.41)	4.33 (1.37)	0.424	0.494
	CANTAB SSP	Sta. Score^b^		−0.54 (0.64)	−1.05 (1.00)	0.257	0.607
	CANTAB SSP	Length^b^		6.53 (1.27)	4.98 (0.79)	0.034	1.466
Executive working memory	CANTAB OTS^k^	Time^b^		27.7 (10.30)	20.2 (4.68)	0.114	0.938
Attention ability	TAP^l^ A. oW	Median^b^		297 (54.0)	303 (49.2)	0.812	0.116
	TAP A. oW	SD^b^		68.0 (32.5)	52.0 (24.5)	0.287	0.556
	TAP A. mW	Median^b^		280 (39.8)	309 (69.0)	0.286	0.515
	TAP A. mW	SD^b^		48.8 (12.8)	53.9 (22.6)	0.566	0.278
	TAP A.	Key value^c^		0.01 (0.090)	−0.02 (0.070)	0.860	0.110
	TAP G.A.	Outlet^b^		1.88 (1.13)	1.88 (1.89)	1.000	0.000
	TAP G/N	Median^b^		613 (105)	607 (52.1)	0.898	0.072
	TAP G/N	Error^c^		2.00 (3.00)	0.00 (1.00)	0.072	0.800
Spatial orientation	DP	2 m^c^		56.0 (36.4)	32.5 (16.8)	0.074	0.830
		2.5 m^b^		55.7 (26.9)	41.0 (29.8)	0.351	0.518
		3 m^c^		94.5 (72.2)	68.0 (56.2)	0.430	0.380
		All conditions^b^		56.0 (36.4)	32.5 (16.8)	0.129	0.874
	Triangle Completion Test	All conditions^c^		152 (44)	109 (26)	0.041	0.890
		Walking					
		All conditions^c^		123 (8)	77.7 (33.2)	0.003	1.200
		Right	60°^b^	123 (191)	73.5 (76.8)	0.049	1.000
			90°^b^	126 (89.2)	64.5 (57.8)	0.082	0.770
			120°^b^	130 (50.5)	96.4 (52.9)	0.189	0.650
		Left	60°^b^	138 (54.2)	60.5 (39.8)	0.009	1.500
			90°^b^	81.5 (66)	66.0 (39)	0.104	0.790
			120°^b^	122 (165)	118 (105.8)	0.151	0.740
		Wheelchair					
		All conditions^b^		197 (68)	149 (42.8)	0.080	0.845
		Right	60°^b^	189 (129)	160 (93.9)	0.569	0.257
			90°^b^	196 (107)	129 (92.9)	0.150	0.669
			120°^b^	166 (109)	180 (93.3)	0.753	0.138
		Left	60°^b^	202 (128)	126 (77.9)	0.129	0.717
			90°^b^	170 (103)	136 (88.3)	0.434	0.584
			120°^b^	257 (108)	164 (70.8)	0.037	1.018
	RM	All conditions^b^		35.5 (11.9)	27.4 (9.25)	0.153	0.760
		One rotation^c^		22.8 (15.2)	24.5 (43.9)	0.713	0.410
		Two rotations^b^		32.2 (18.3)	44.3 (20.2)	0.264	0.628
		Four rotations^b^		41.1 (25.9)	26.3 (16.9)	0.200	0.677
		Eight rotations^b^		43.2 (24.3)	37.4 (24.6)	0.641	0.237
	C-Screen	Total output^b^		18.0 (42.2)	38.1 (26.8)	0.531	0.569

Eleven patients and eleven controls. For normally and homogeneously distributed data, the unpaired *t*-test and for not normally and not homogeneously distributed data the Mann–Whitney U-test (with the Bonferroni adjustment) was used.

AVLT; Digit span memory test; Stroop effect (subtest 1–3); RWT; CANTAB (OTS; SSP); TAP (A, alertness; oW, without warning signal; mW, with warning signal, G.A., divided attention; G/N, go/nogo). Cognitive testing—spatial orientation. DP; Triangle Completion Test; RM and C-Screen.

^a^ Rey Auditory Verbal Learning Test.

^b^ Mean and standard deviation.

^c^ Median and interquartile range.

^d^ Regensburg Word Fluency Test.

^e^ Formal lexical.

^f^ Semantic.

^g^ Formal lexical category switching.

^h^ Semantic category switching tasks.

^i^ Cambridge Neuropsychological Test Automated Battery.

^j^ Spatial Span.

^k^ One Touch Stockings.

^l^ Test Battery for Attention Assessment.

### Cognitive testing—spatial orientation

**Figure 3:**
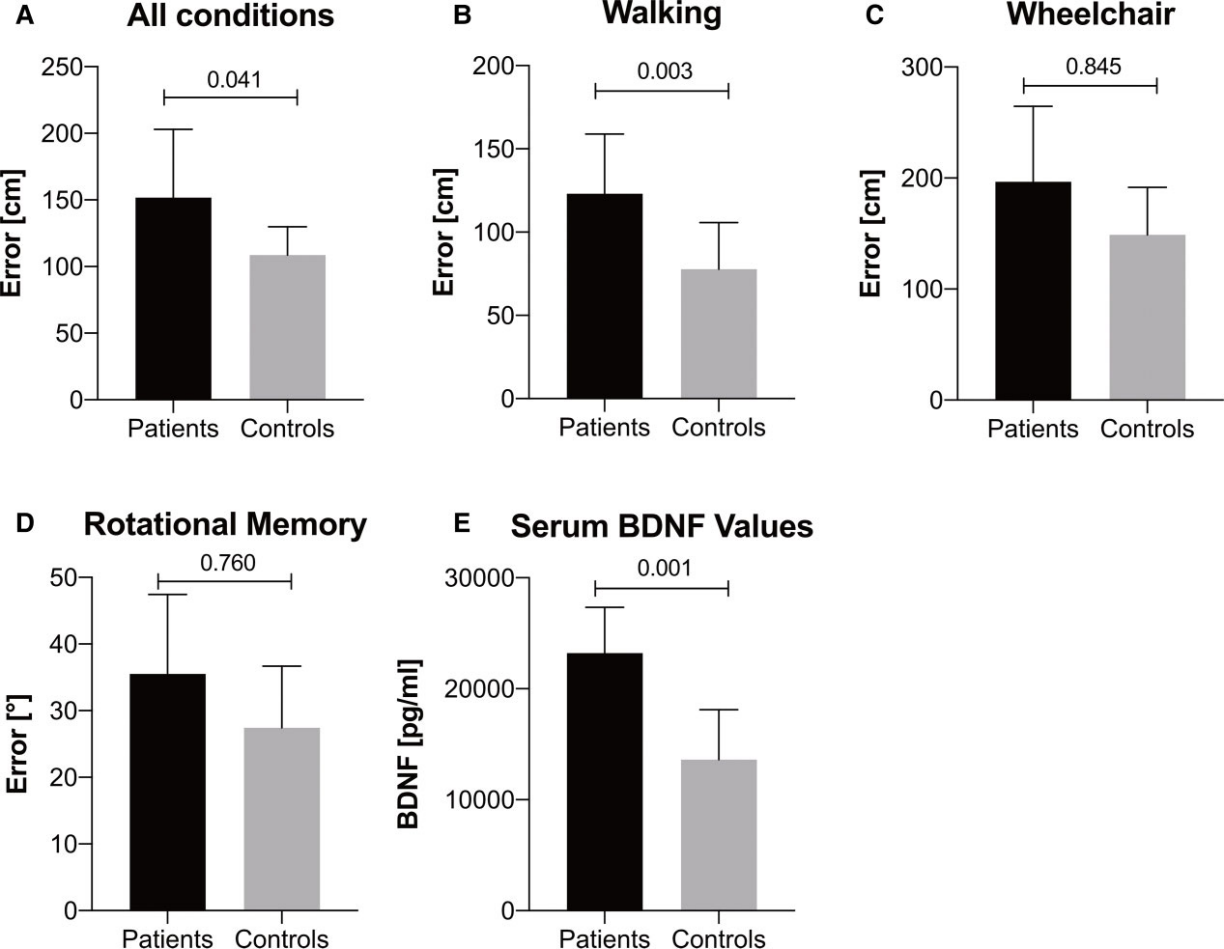
**Error on TCT, RM and serum BDNF.** Error on TCT on all conditions (**A**; *P *= 0.041, *d* = 0.890, *n* = 20); Mann–Whitney U-test, walking (**B**; *P *= 0.003, *d* = 1.200, *n* = 20); Mann–Whitney U-test, wheelchair (**C**; *P *= 0.080, *d* = 0.845, *n* = 20); unpaired *t*-test and on RM on all conditions (**D**; *P* = 0.153, *d* = 0.760, *n* = 16); unpaired *t*-test for both patients and controls. Serum BDNF values in patients and controls (**E**; *P *= 0.001, *d* = 2.040, *n *= 18); unpaired *t*-test.

MG patients showed significant lower performance in somatosensory-related spatial orientation (walking, all conditions, *P *= 0.003, *d* = 1.200), but not in vestibular-related spatial orientation (wheelchair, all conditions, *P *= 0.080, *d* = 0.845). Additionally, no significant differences in vestibular-related RM tests were observed (all conditions, *P* = 0.153, *d* = 0.760) (Table 3 and Fig. 3).

### Brain-derived neurotrophic factor

Serum BDNF levels showed significant higher values in MG patients compared to healthy controls (MG patients: 24 162 ± 4148; healthy controls: 14 257 ± 5474; *P *= 0.001, *d* = 2.040; Fig. 3).

### Magnetic resonance imaging

**Figure 4:**
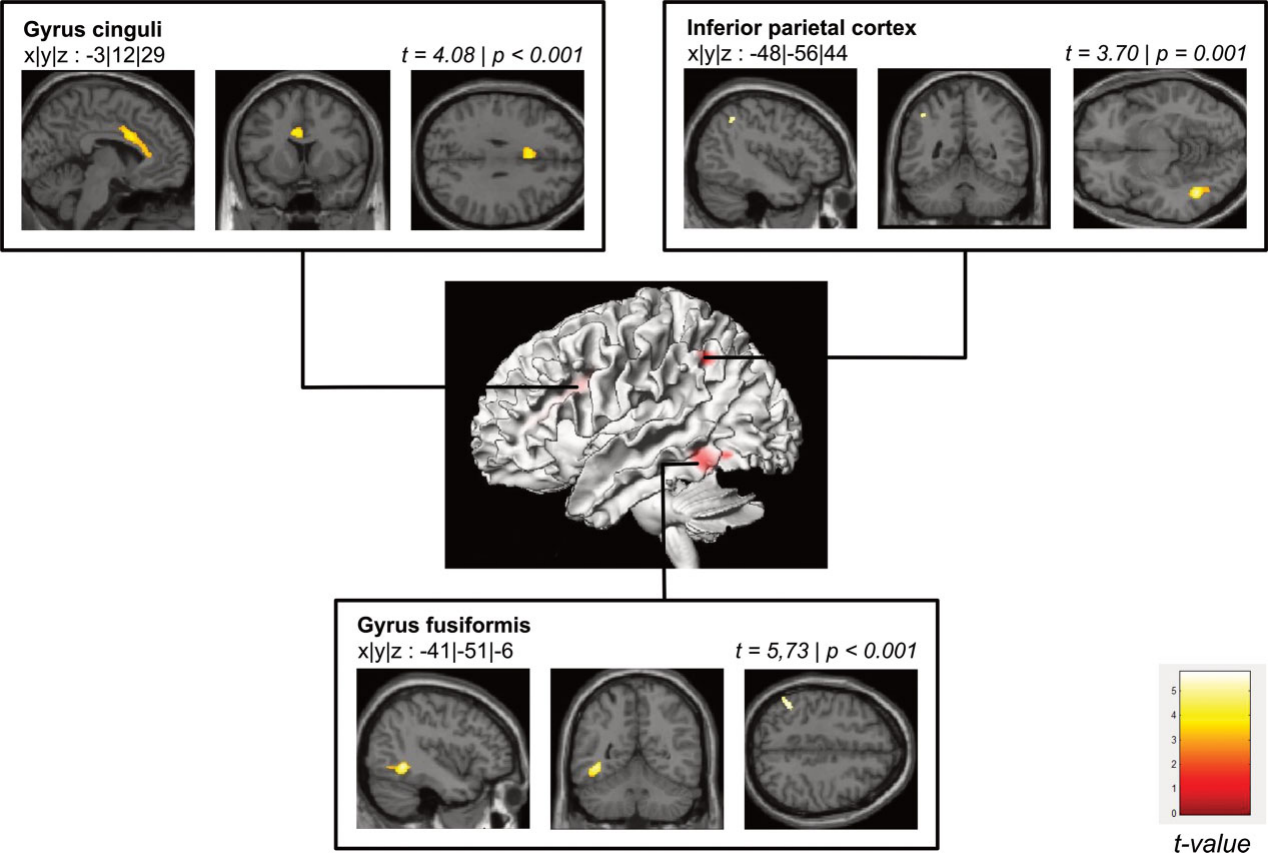
**Grey matter volume changes.** Significant lower grey matter volumes in patients compared to the healthy controls. Montreal Neurological Institute coordinates, statistical parameters and hemisphere of the significant volume differences.

Using VBM, significant lower grey matter volumes were observed in MG patients in the left fusiform gyrus (*P *≤ 0.001, *t* = 5.73), in the left cingulate gyrus (*P *≤* *0.001, *t* = 4.08) and in the left inferior parietal cortex (*P *= 0.001, *t* = 3.70, Fig. 4).

### Correlation analysis

The correlation analysis in MG patients between significant regional brain region changes, BDNF levels and behavioural tests did not reveal any associations in MG patients (Table 4).

**Table 4:** Correlation analysis

Region/condition	Test	Cor	95% CI	*t*	*df*	*P*-value
Gyrus cinguli	AVLT^a^	0.046	−0.417; 0.490	0.191	17	0.851
Gyrus cinguli	SSP^b^	−0.413	−0.845; 0.346	−1.20	7	0.270
IPL	TCT^c^	0.293	−0.257; 0.700	1.107	13	0.288
IPL^d^	AVLT	0.240	−0.240; 0.626	1.020	17	0.322
BDNF^e^	AVLT	−0.336	−0.703; 0.172	−1.383	15	0.187
BDNF	SSP	0.381	−0.379; 0.834	−1.090	7	0.312
BDNF	TCT	0.375	−0.194; 0.756	1.403	12	0.186

Gyrus cinguli and AVLT (total output), Gyrus cinguli and SSP; IPL and spatial navigation (TCT; walking all conditions); IPL and AVLT (total output); BDNF serum and AVLT (total output); serum BDNF and SSP (length), serum BDNF and spatial navigation (TCT; walking all conditions).

^a^ Rey Auditory Verbal Learning Test.

^b^ Spatial Span.

^c^ Triangle Completion Test.

^d^ Inferior parietal lobe.

^e^ Brain-derived neurotrophic factor.

## Discussion

The aim of this study was to investigate the structural (grey matter brain volumes) and the functional (cognitive with a focus on spatial orientation, and BDNF) brain changes in MG patients compared to matched healthy controls. The present results confirm our hypotheses, that structural and functional brain may changes occur in MG.

MG patients showed significant deficits in visuospatial working memory, verbal learning and non-visual somatosensory-dependent spatial orientation, together with lower grey matter volumes in the cingulate gyrus, in the inferior parietal lobe (IPL) and in the fusiform gyrus. Furthermore, MG was related to significantly elevated serum BDNF levels.

### Cognitive deficits in MG patients—general

We observed especially lower test scores of verbal and spatial functions. In questionnaires on dementia, depression and fatigue, our MG patients showed no significant difference compared with healthy controls. These findings are consistent with previous results. Paul *et al.*^36^ also demonstrated significant lower performances in reaction speed, verbal and visual learning measures, and information processing in MG patients. They could show that these cognitive deficits were not associated with mood disorders, disease duration or medication use. The results of Marra *et al.*^37^ are consistent with part of our outcomes. No significant deficits were found in MG patients in tests such as the MMSE and depression. However, in contrast to our results, they also reported no significant deficits in working memory tests and word fluency test.^6^ Iwasaki *et al.*^38^ reported significantly lower MMSE scores in MG patients in their study, in contrast to our results. Likewise, Drozdick *et al.*^39^ found significantly lower results of the MG patients in the ‘Wechsler Memory Scale’. The other cognitive parameters examined, however, showed no significant differences between the MG patients and healthy controls. Therefore, most (including the present one) but not all studies support that general cognitive abilities are intact in MG.

### Cognitive deficits—spatial orientation

Based on our hypothesis of sensorimotor deprivation in MG, we especially expect deficits in functions that rely on this input, namely spatial orientation. In accordance with our prediction, MG patients showed significantly reduced results in somatosensory-related spatial navigation, namely the walking condition of the TCT. In contrast, spatial navigation abilities that rely on vestibular input (wheelchair condition of the TCT, rotation memory task) revealed no group differences.^32^ Vestibular function, thus, can be assumed to be intact in MG.

### Grey matter brain changes in MG

Significant lower grey matter brain volumes were observed in three areas in MG patients compared to healthy controls: in the cingulate gyrus, the IPL and the fusiform gyrus.

#### Cingulate gyrus

The cingulate gyrus is functionally part of the limbic system and is, together with the cerebellum and other regions, involved in sensomotoric functions.^40,41^ Since MG patients are on average less able to perform coordinative movements due to load-dependent paralysis,^41^ this may explain the smaller volume in this brain region. Furthermore, the cingulate gyrus is part of the Papez circuit and associated with memory functions.^42^ As reported above, our MG patients showed impairments in these functions. Hence, these cognitive deficits may be related to the patients’ smaller cingulate gyrus volumes. However, no correlation between brain volumes and behaviour was observed. One potential explanation could be our small sample size.

#### Inferior parietal lobe

In line with the assumption of peripheral deprivation, we found significantly smaller grey matter brain volumes in the inferior parietal cortex in MG patients. The inferior parietal cortex is associated with sensory projection centres, goal-directed motor behaviour as well as working memory.^43–45^ For example, Baldo and Dronkers^46^ found that patients with focal lesions in the IPL showed disproportionately impaired span, rhyme and repetition performance, hence, a phonological memory deficit. Thus, it can be hypothesized that the volume reduction in the IPL in our MG patients led to both impaired verbal learning performance and deficits in somatosensory-dependent spatial orientation.

#### Fusiform gyrus

The fusiform gyrus in the temporal lobe is associated with multisensory object perception and tactile recognition.^47–50^ Previous studies have shown larger grey matter volume in the fusiform gyrus following increased sensory input, counter wise, significant reductions in grey matter volume in the fusiform gyrus in our MG patients may be attributed to the lack of somatosensory input.

### Brain-derived neurotrophic factor

BDNF is a synaptic growth factor that is secreted in response to excitatory synaptic activity and serves as a key mediator of neuroplasticity in the brain, including somatosensory and visual cortex.^51–56^ BDNF promotes neurogenesis and synaptic plasticity and its expression and release can be decreased by various factors, such as pro-inflammatory cytokines, advanced age, chronic stress, mental and neurodegenerative diseases.^57–63^

Our results showed significantly higher BDNF serum levels in MG patients. Potential mechanisms of increased BDNF levels could be a BDNF synthesis of (neuro-)inflammatory cells or a protective mechanism against progressive loss of neuromuscular junctions and/or drug treatment with cholinesterase inhibitors. Already da Penha Berzaghi *et al.*^64^ showed an increase in hippocampal BDNF levels in rats after treatment with direct parasympathomimetics (pilocarpine) which serve a neuroprotective role during inflammation.^65–68^ This correlates in humans, with the reported almost four times higher BDNF serum levels in LOMG than in early-onset MG (EOMG).^69^ In addition, pro-inflammatory cytokines reduce BDNF gene expression.^58^ Since LOMG is associated with less inflammation than EOMG, this could also be a possible link.

### Limitations and perspectives

The present results provide the first major insight into the structural and functional changes of the brain in MG patients. Thus, a new line of research has been illuminated, which can be the basis for further studies. The main limitation of the current study is its small sample size, which may be the cause of a lacking correlation between functional and structural brain data, the all-male subject group, as well as the fact that MR volume differences were seen only on the uncorrected level. Future studies should investigate structural and functional brain changes (i) in myasthenia gravis in a larger cohort, (ii) in a long-term study and (iii) in further neurologic disease associated with sensorimotor deprivation (e.g. Guillain Barre Syndrome, chronic inflammatory demyelinating polyradiculoneuropathy). Additionally, gender aspects should be considered in future studies.

Based on these preliminary results indicating structural and functional brain changes in MG, future studies could investigate the potential impact of physical exercise interventions to counteract these changes.

## Abbreviations

AVLT =: Rey Auditory Verbal Learning Test
BDI =: Beck’s Depression Inventory
BDNF =: brain-derived neurotrophic factor
CANTAB =: Cambridge Neuropsychological Test Automated Battery
DP =: Distance Perception
EOMG =: early-onset myasthenia gravis
IPL =: inferior parietal lobe
LOMG =: late-onset myasthenia gravis
MG =: myasthenia gravis
MMSE =: Mini Mental State Examination
MRC =: Medical Research Council
OTS =: One Touch Stockings
RM =: Rotational Memory
RWT =: Regensburg Word Fluency Test
SSP =: Spatial Span
TAP =: Test Battery For Attention Assessment
TCT =: Triangle Completion Test
VBM =: voxel-based morphometry
